# Engineered 3D-printed artificial axons

**DOI:** 10.1038/s41598-017-18744-6

**Published:** 2018-01-11

**Authors:** Daniela Espinosa-Hoyos, Anna Jagielska, Kimberly A. Homan, Huifeng Du, Travis Busbee, Daniel G. Anderson, Nicholas X. Fang, Jennifer A. Lewis, Krystyn J. Van Vliet

**Affiliations:** 1 Department of Chemical Engineering, Massachusetts Institute of Technology, Cambridge, MA 02139 USA; 2 Biosystems & Micromechanics Interdisciplinary Research Group (BioSyM), Singapore-MIT Alliance in Research & Technology (SMART), Singapore, Singapore; 3 Department of Materials Science and Engineering, Massachusetts Institute of Technology, Cambridge, MA 02139 USA; 4 Wyss Institute for Biologically Inspired Engineering, Cambridge, MA 02138 USA; 5 School of Engineering and Applied Sciences, Harvard University, Harvard, MA 02138 USA; 6 Department of Mechanical Engineering, Massachusetts Institute of Technology, Cambridge, MA 02139 USA; 7 David H. Koch Institute for Integrative Cancer Research, Massachusetts Institute of Technology, Cambridge, MA 02139 USA; 8 Institute for Medical Engineering and Sciences, Massachusetts Institute of Technology, Cambridge, MA 02139 USA; 9 Harvard-MIT Division of Health Sciences & Technology, Cambridge, MA 02139 USA; 10 Department of Biological Engineering, Massachusetts Institute of Technology, Cambridge, MA 02139 USA

## Abstract

Myelination is critical for transduction of neuronal signals, neuron survival and normal function of the nervous system. Myelin disorders account for many debilitating neurological diseases such as multiple sclerosis and leukodystrophies. The lack of experimental models and tools to observe and manipulate this process *in vitro* has constrained progress in understanding and promoting myelination, and ultimately developing effective remyelination therapies. To address this problem, we developed synthetic mimics of neuronal axons, representing key geometric, mechanical, and surface chemistry components of biological axons. These artificial axons exhibit low mechanical stiffness approaching that of a human axon, over unsupported spans that facilitate engagement and wrapping by glial cells, to enable study of myelination in environments reflecting mechanical cues that neurons present *in vivo*. Our 3D printing approach provides the capacity to vary independently the complex features of the artificial axons that can reflect specific states of development, disease, or injury. Here, we demonstrate that oligodendrocytes’ production and wrapping of myelin depend on artificial axon stiffness, diameter, and ligand coating. This biofidelic platform provides direct visualization and quantification of myelin formation and myelinating cells’ response to both physical cues and pharmacological agents.

## Introduction

Myelination is a key developmental milestone in vertebrate neuronal function, and compromised myelin sheath formation or repair is a hallmark of several central nervous system (CNS) diseases^1–3^. Glial cells such as oligodendrocytes produce and wrap the protective and insulating lipid-rich myelin membrane around axons of neurons. *In vitro* models and materials to understand and promote this interaction among glial cells and neurons are of both scientific and technological interest. Several models have been used extensively, but typically compromise between complexity of the three-dimensional *in vivo* tissue microenvironment^4^ (e.g., organotypic tissue slices, co-cultures) and high-throughput feasibility for screening, imaging and interpretation (e.g., transparent, flat, stiff materials). While co-cultures^5^ have been recently developed for high-throughput drug screening, inclusion of both neurons and oligodendrocytes confers challenges in image-based quantification of myelination, incurs lengthy protocols and appreciable cost with limited reproducibility, and holds potential for off-target and cell type cross-talk that complicate interpretation of mechanism for the cell types of interest^4,6^.

Recently, more reductionist approaches that mimic key features of neuronal axons have been explored, each with its advantages and limitations. Materials ranging from chemically fixed (i.e., crosslinked) cells^7^ to heavily crosslinked polymers^8,9^ carbon ^10^ and glass^11–13^ have been used to decouple molecular cues from biophysical properties and to screen drug effects on myelin wrapping by oligodendrocytes and Schwann cells. Bullock *et al*.^11^ and Howe^12^ first considered randomly oriented, glass microfibers as axon surrogates; Howe demonstrated that extent of wrapping by oligodendrocytes varied with fiber coating. Rosenberg *et al*. later showed that intact, chemically fixed axons enabled compact, concentric and multilaminar myelination, suggesting that dynamic axonal signaling is not required to initiate or complete ensheathment^7^. Lee *et al*.^8^ and Bechler *et al*.^9^ used electrospun fibers as axon mimics to decouple molecular cues from the biophysical property of axon diameter and observed preferential myelin wrapping around fibers of larger diameter. Mei *et al*.^13^ developed fused-silica cones, which could be viewed in-plane for imaging and screening of drug effects on oligodendrocyte production of engaged myelin. While providing the potential for rapid comparative analysis of various conditions on myelin production, the patterned glass cones did not present the cylindrical geometry of biological axons, and the multilayered membrane compaction considered a key feature of myelination was not reported. Merolli *et al*.^10^ extended the concept of axon mimics to human Schwann cells, demonstrating direct observation of cells’ interactions with a single carbon microfiber. All of those prior models neglected a key feature of brain tissue and specifically of neuronal axons: extremely low mechanical stiffness. Nervous tissue is among the most compliant of the biological “soft tissues” with Young’s elastic modulus *E* ~ 0.1–1 kPa^14–16^, which is approximately six orders of magnitude lower than silica glass^17^ or tissue culture polystyrene^18^ used typically for neurological cell culture. Glial cell lineages are mechanosensitive: mechanical cues modulate biology of astrocytes^19,20^, microglia^21^, Schwann cells^22^ and oligodendrocytes^23–31^. We have shown that material stiffness modulates proliferation, migration, and differentiation of oligodendrocyte progenitor cells (OPC) into myelinating oligodendrocytes^24^. Furthermore, local reduction in tissue stiffness reported in neurodegenerative disorders such as Alzheimer’s and multiple sclerosis, also characterized by inflammation and decreased myelin matter^32,33^, may be an important factor affecting oligodendrocyte ability to repair myelin in these pathological environments. Therefore, it is critical that studies of myelination in healthy and pathological contexts are conducted in mechanically relevant environments.

Here we developed arrays of artificial axons that provide independent control of fiber geometry, mechanical stiffness, and ligand functionality to replicate key features of biological axons in health and disease. We aimed specifically to produce cylindrical, freestanding fiber lengths of low mechanical stiffness reflecting that of biological axons. This approach considers key physical, mechanical, and surface properties of axons, rather than the electrophysiological function of electric conductivity that is explored in other models^34^. Using multiple additive manufacturing methods, polymers, and architectures, we demonstrate engineered microenvironments that uniquely approach biological axon-level stiffness, diameter and spacing. We show that these 3D-printed polymer fibers are compatible with oligodendrocyte adhesion and engagement *in vitro*. This approach facilitates direct observation of myelin-rich wrapping, and quantitative comparison of artificial axon features that promote this critical interaction with oligodendrocytes.

## Results

### Design of artificial axons

**Figure 1:**
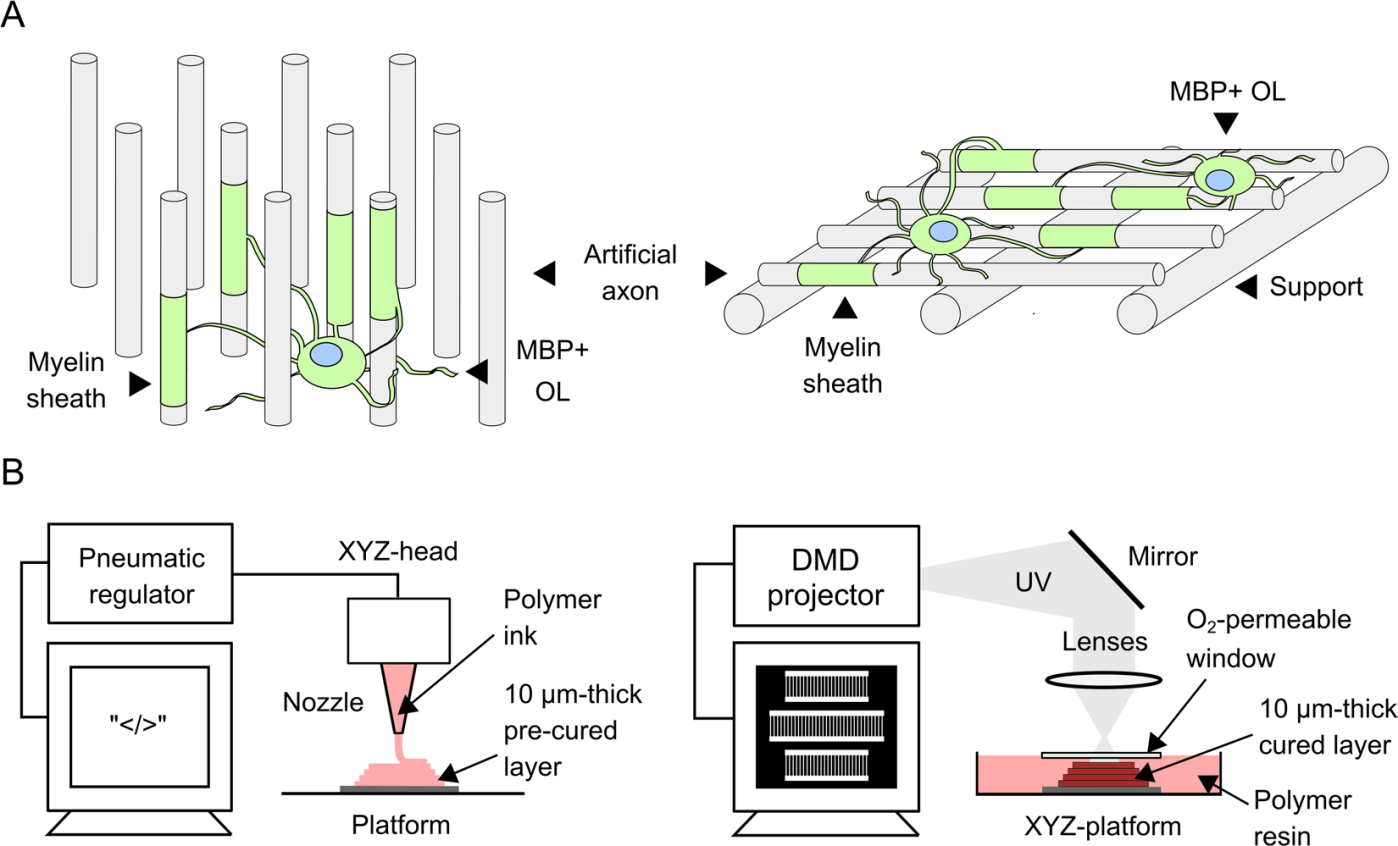
Schematic of artificial axons and additive manufacturing approaches. (**A**) Arrays of vertical fibers (left) of uniform diameter in close proximity resemble geometry of neuronal axon bundles and white matter tracts, and enable complete wrapping around fiber circumference, while allowing fast detection of concentric myelin. Suspended horizontal fibers (right) allow high throughput acquisition of myelin segment length. (**B**) Direct ink printing (left): inks are extruded through a nozzle on a translational head to fabricate predefined three-dimensional constructs. Projection micro-stereolithography (right): slices of computer-aided design (CAD) models are sequentially sent to a digital micromirror device (DMD) illuminated by a light source, and projected sequentially onto a photopolymer resin bath.

Figure 1 demonstrates our approach schematically, guided by mechanical and morphological features of axon tracts in the CNS. Axons are approximately cylindrical projections extending from neuron bodies; axons lie in close proximity in such tracts, but are sufficiently freestanding to support wrapping of myelin by multiple oligodendrocytes. In white matter tracts, axon diameters vary from ~0.2 to 9 µm with a median diameter of ~0.6 µm^35^. Mechanical characterization of brain matter and single cells suggests that neurons may be approximated as elastic solids of *E* ranging 10^2^–10^3^ Pa^14^, which is much lower than that of most 3D printed thermoplastics or glasses of *E *~ 10^7^–10^10^ Pa^17,18,36–38^.

### Additive manufacturing methods for artificial axons

**Figure 2:**
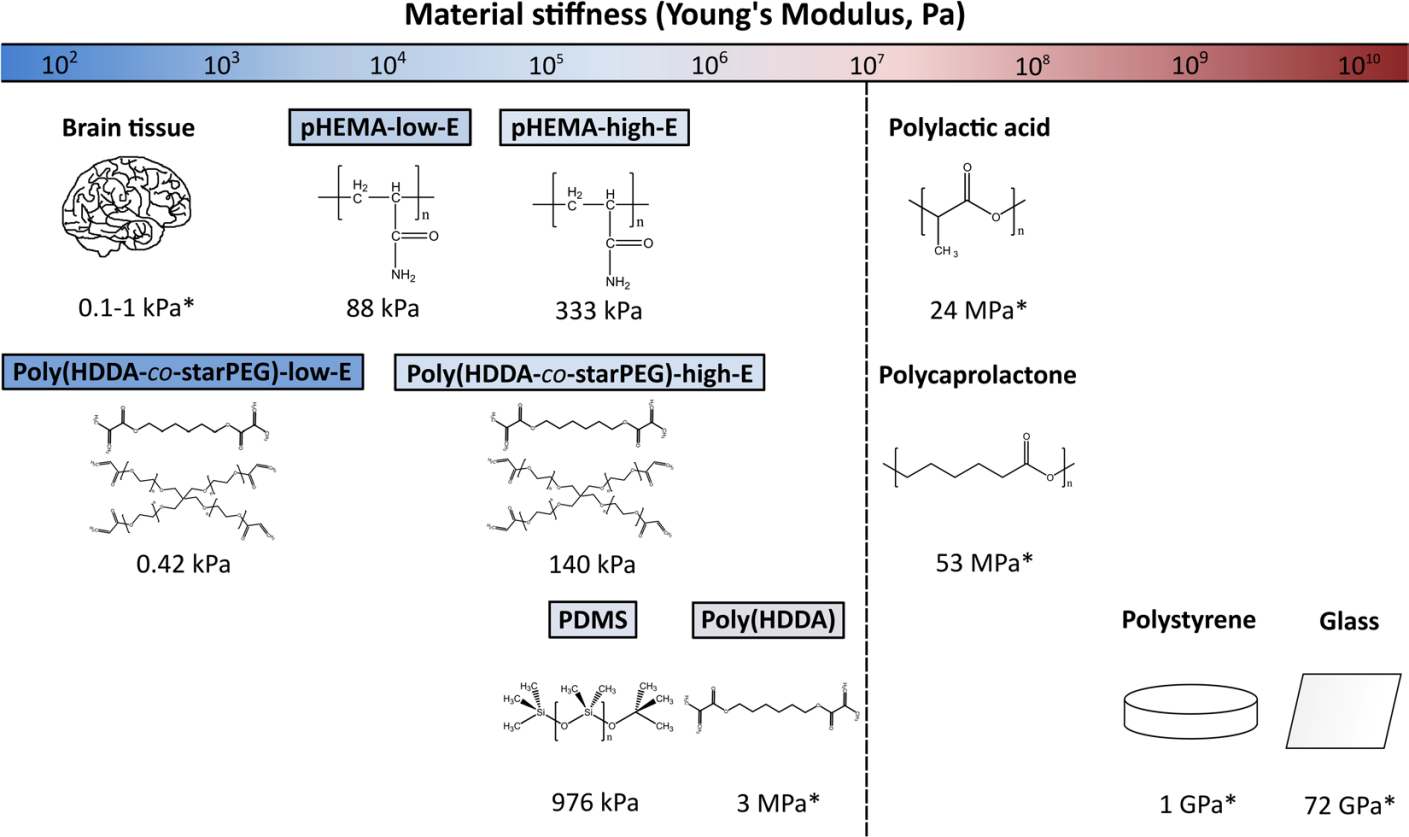
Materials for additive manufacturing of artificial axons (left, Young’s elastic modulus < 10^7^ Pa) afford mechanical stiffness more similar to CNS cells and tissues than materials used currently for myelination assays (right, Young’s elastic modulus >10^7^ Pa); *reported literature values from refs^14–16,36–39,44^. The Young’s elastic moduli of artificial axon materials were determined by means of atomic force microscope-enabled nanoindentation (see Materials and Methods).

**Figure 3:**
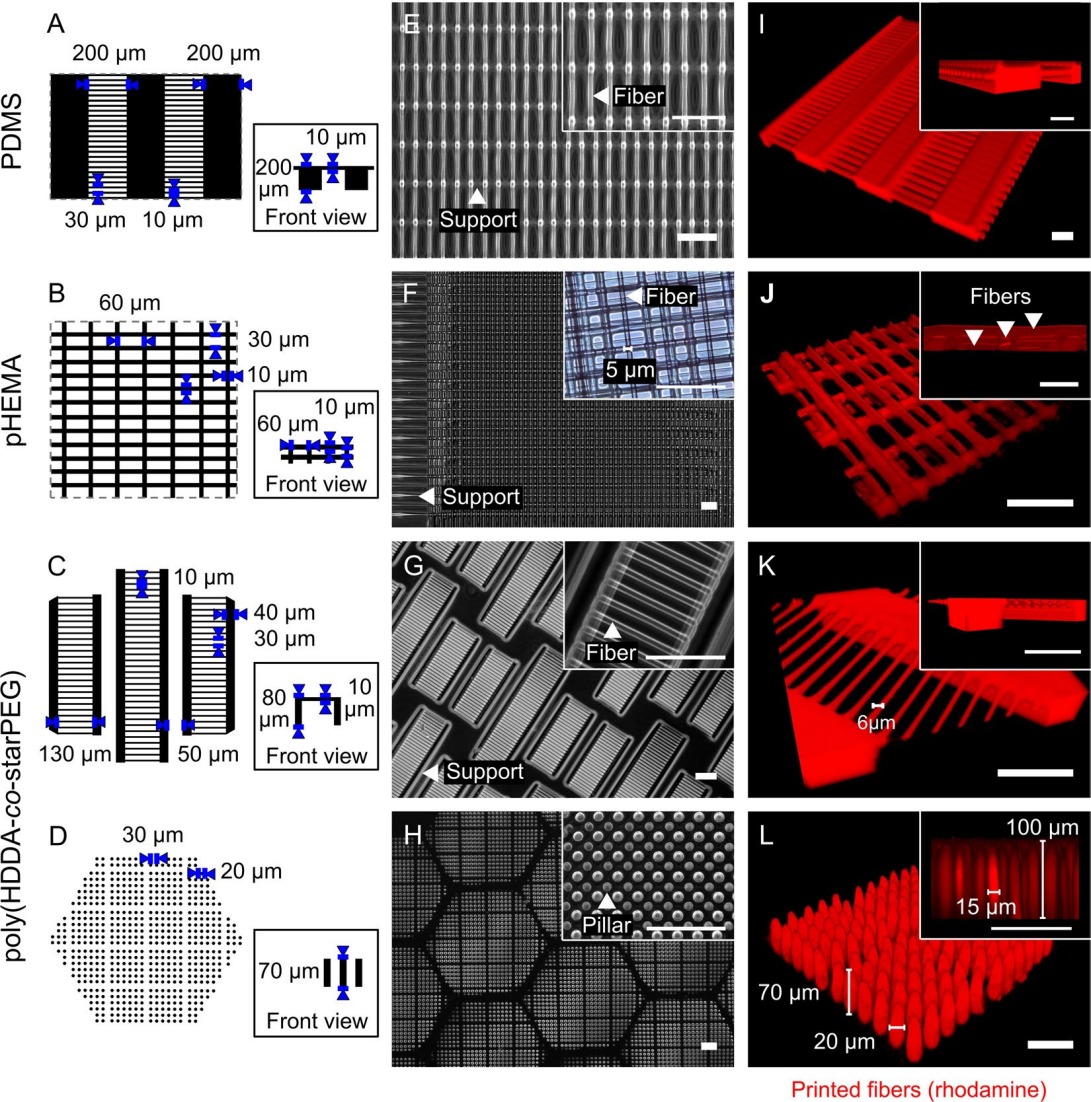
Fabrication of artificial axons with two different additive manufacturing approaches. Schematic of (**A**) PDMS and (**B**) pHEMA fiber bundles with fiber diameter 10 µm and lengths 30–200 µm, fabricated via direct ink printing. (**C**,**D**) CAD-generated digital masks of fiber modules of predetermined diameters 10–20 µm and lengths 70–130 µm, fabricated by projection micro-stereolithography, or PμSL. (**E**–**H**) Fabricated fibers examined by phase contrast; (**I**–**L**) three-dimensionality and uniformity evaluated with confocal microscopy. Printed fiber and pillar diameters ranged from 5 µm (**F**) to 20 µm (**L**). Pillar arrays in (**L**) and (**L** insert) of nominal pillar diameter 20 µm and 15 µm, respectively. Scale bars are 100 µm.

Although lithography-based techniques enable fabrication of microscale high aspect ratio features, those patterned materials exhibit high mechanical stiffness (*E* > MPa). Creating mechanically compliant (*E* < MPa) unsupported features is particularly challenging due to coupling of low elastic modulus, demolding mechanics and operating conditions that often induce structural collapse and deformation^39^. Direct ink writing and projection micro-stereolithography (PµSL) offer the unique advantages of fabricating programmably defined 3D microstructures in a semi-high throughput manner, with high vertical aspect ratios, overhanging parts, and flexibility of printing both elastic and viscoelastic materials (Fig. 1B)^40–42^. We adapted both additive manufacturing or 3D printing technologies and established a library of biocompatible polymers spanning a wide range of mechanical properties (Fig. 2) to print arrays of artificial axons with diameters <10 µm in vertical (pillars) and horizontal (fibers) orientations, unprecedented mechanical compliance (*E* < 1 kPa), and high aspect ratios of appreciable unsupported length spans (Fig. 3).

### Materials for artificial axons

Several polymer inks have been produced for direct writing^40,41,43^. In this study, we optimized two types of inks for artificial axon production: polydimethylsiloxane (PDMS) inks that form elastic and deformable fiber arrays with *E* = 976 ± 11 kPa (Fig. 3A,E,I) and two pHEMA-based inks^41^ that form viscoelastic hydrogels after hydration of distinguishable stiffness: low *E* = 88 ± 10 and high *E* = 333 ± 30 kPa (Fig. 3B,J). The Young’s elastic moduli *E* of the fiber materials were determined by means of atomic force microscope-enabled nanoindentation (see Materials and Methods, AFM-enabled nanoindentation and rheology, for characterization details). We also developed two poly(HDDA-*co-*starPEG)^38^ resins of distinguishable stiffness: high *E* = 140 ± 35 kPa and very low *E* = 0.42 ± 0.14 kPa, to achieve the desired printing, cell compatibility and elastic properties via PµSL (Fig. 3C,D,G,H,K,L). (See Materials and Methods for specific composition and processing parameters for each polymer type and printing method.) Copolymerization of HDDA^38,44^ with traditionally biocompatible and compliant PEG polymer precursors mitigates the challenges that preclude HDDA implementation in biological applications while retaining capabilities for PµSL fabrication^38^ (Supplementary Fig. S1). Importantly, these polymer compositions and methods facilitated fabrication of compliant fibers matching the range of biological axon stiffness in the format of unsupported spans, a feature required to facilitate observations of wrapping. The Young’s elastic moduli of printed fibers ranged 10^2^–10^6^ Pa, which is orders of magnitude lower than state-of-art polymers used in glial cell cultures (e.g., polystyrene, polycaprolactone, or polylactic acid of 10^7^–10^9^ Pa)^17,18,36,37^.

### OPCs adhere and migrate along artificial axons

**Figure 4:**
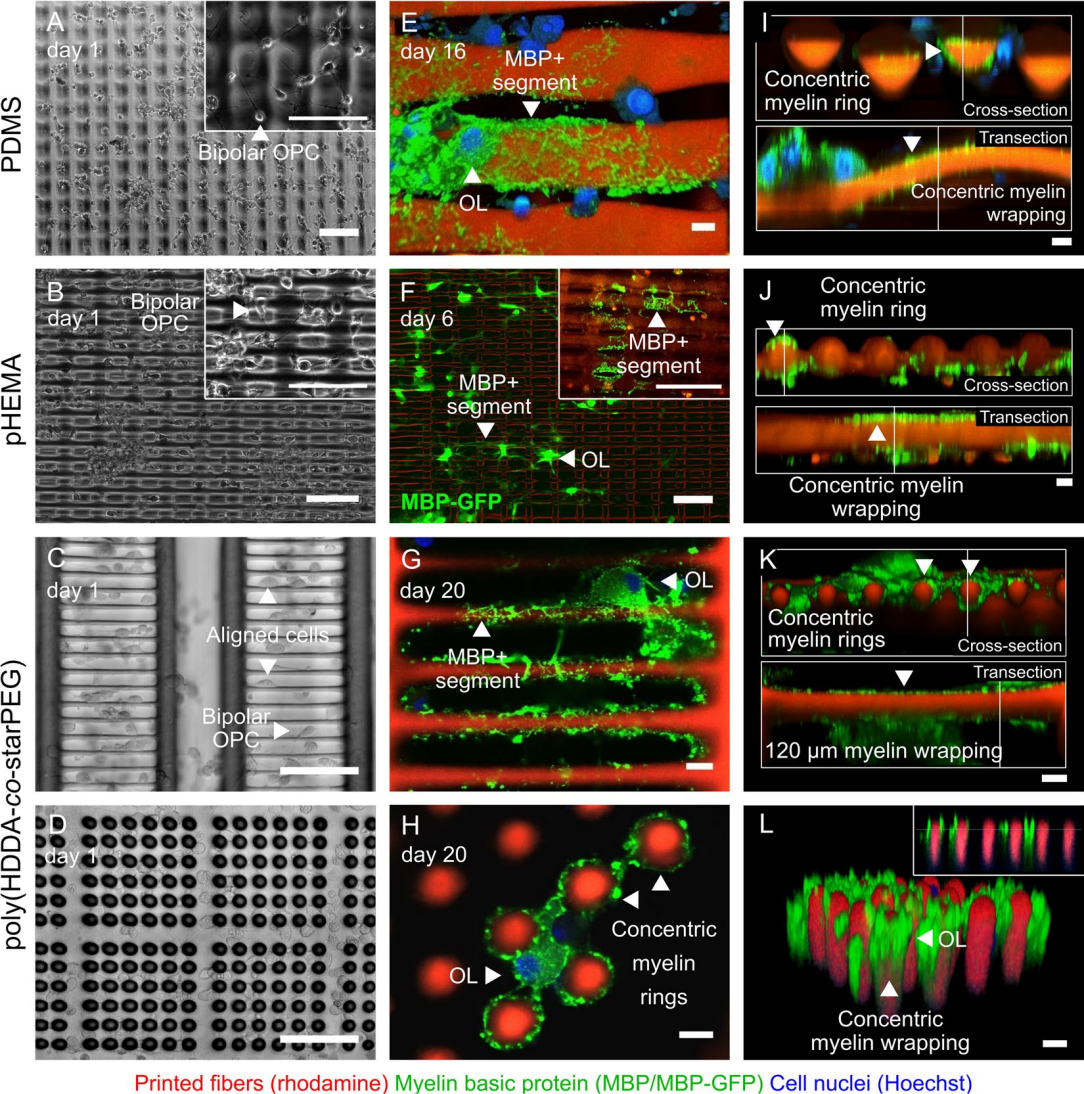
Oligodendrocytes adhere to, differentiate, and produce myelin basic protein (MBP)-positive membrane that wraps artificial axons. (**A**–**D**) OPC engagement, migration and proliferation at day 1 after seeding on artificial axons with diameters 10–20 µm; fibers were functionalized with (**A**) fibronectin, (**B**–**E**, **F**-insert, **G**–**L**) laminin or (**F**) PDL. (**E**–**L**) Oligodendrocyte differentiation and MBP-positive membrane wrapping of artificial axons; (**E**–**H**) plan views; (**I**–**L**) cross- and transections showing MBP-positive membrane (green) around the artificial axon fibers (red); leftmost column scale bars = 100 µm; center and rightmost column scale bars = 10 µm. The corresponding Young’s moduli are *E* = 976 ± 11 kPa for PDMS; *E* = 333 ± 30 kPa for pHEMA; *E* = 140 ± 35 kPa for poly(HDDA-*co-*starPEG) in (**C**, **G**, and **K**); and *E* = 0.42 ± 0.14 kPa for poly(HDDA-*co-*starPEG) in (**D**, **H** and **L**).

Maturation of oligodendrocyte progenitor cells to myelinating oligodendrocytes requires that OPCs migrate toward and engage axons *in vivo*, ultimately differentiating to oligodendrocytes that wrap around, and ensheath the axon in myelin membrane that extends over 10 s of micrometers along the axon length. We functionalized artificial axons with laminin, a known regulator of myelination via integrin interactions that is expressed on the axon surface^45^, or fibronectin, as an alternative integrin-binding ligand. (We note that there is no supporting evidence of fibronectin expression on CNS axons). Laminin and fibronectin are also components of the CNS extracellular matrix. We used a nonspecific adhesion promoter, poly-D-lysine, as a control (see Materials and Methods, Artificial axon functionalization). We then monitored cell migration, engagement with artificial axons, and differentiation. OPCs adhered to artificial axons comprising all tested combinations of fiber material and coating (i.e., PDMS with laminin, fibronectin and PDL; pHEMA with laminin, fibronectin and PDL; poly(HDDA-*co*-starPEG) with laminin and PDL) and displayed the bipolar morphology that is a marker of the progenitor stage (Fig. 4 and Supplementary Figs S2, S7). We observed qualitatively that there existed more bipolar cells on fibers coated with laminin or fibronectin, as compared to those coated with PDL (Supplementary Fig. S2). We also recorded cell migration along pHEMA artificial axons with time-lapse imaging (Supplementary Movie S1), demonstrating direct observation of this key property of oligodendrocyte progenitors.

### OPCs differentiate and wrap artificial axons

Within two days in differentiation medium within these artificial axon arrays, OPCs acquired multipolar morphology and continued to mature for at least 20 days. Cell processes engaged multiple adjacent fibers and pillars (Fig. 4E–H, Supplementary Fig. S7, Movies S2 and S3), and cell somas often spanned the empty space between parallel artificial axons (Supplementary Fig. S2). Some cells extended processes to fibers located up to 120 µm from the cell body (Supplementary Fig. S2). We readily detected concentric wrapping of membranes around the artificial axon perimeter and extending along the fiber length with immunostaining for myelin markers such as myelin basic protein (MBP) around Rhodamine-B stained fibers (Fig. 4I–L). Fully wrapped segments ranged in length span from <10 µm to the entire artificial axon length (70–120 µm), as quantified by confocal microscopy and image analysis (Fig. 4K). We also demonstrated that imaging of MBP-positive membrane deposition can be recorded to gain insight on dynamics of myelin formation (Supplementary Movies S4 and S5).

### 3D printing enables axon feature variation to study cell responses

**Figure 5:**
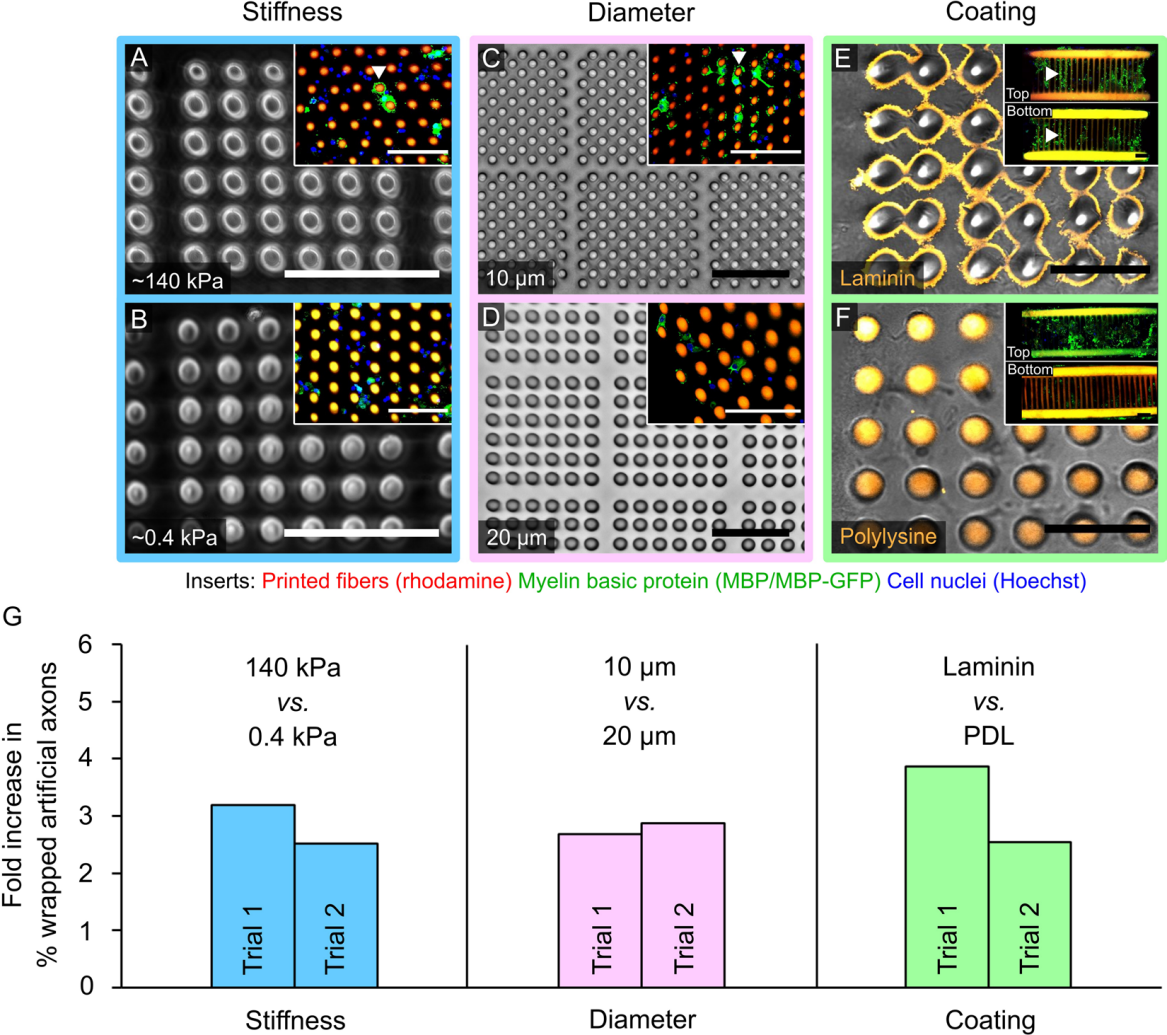
Features of artificial axons can be varied with high precision to interrogate impact of individual cues on cell behavior. (**A**,**B**) Mechanical stiffness of poly(HDDA-*co*-starPEG) pillars varied by changing polymer composition, while maintaining pillar diameter of ~16 μm, aspect ratios of 3:1, and laminin coating for (**A**) *E ~* 140 kPa and (**B**) *E* ~ 0.4 kPa; scale bars = 100 μm. (**C**,**D**) Poly(HDDA-*co-*starPEG) pillar diameter was varied readily with mask modifications while maintaining resin composition and stiffness of 0.4 kPa constant; (**D**) pillar diameter 10 μm, (**E**) pillar diameter 20 μm; scale bars = 100 μm. (**E**,**F**) Poly(HDDA-*co-*starPEG) fibers were sufficiently hydrophilic^38^ to enable high extent of physisorption of common charged ligands including (**E**) laminin and (**F**) poly-D-lysine that persisted for at least 20 days in culture, confirmed with fluorescently labeled laminin and poly-D-lysine, respectively; (**E,F**) insert fiber stiffness ~140 kPa, fiber diameter 10 μm; scale bars = 50 μm. (**G**) Quantification of artificial axon wrapping in response to changes in artificial axon stiffness (blue), diameter (pink), or coating (green). For each of the three comparisons in (**G**), the fold-increase was quantified as the percentage of wrapped artificial axons within the array comprising stiffer, smaller diameter, or laminin-coated fibers, relative to the percentage of wrapped artificial axons within the array comprising compliant, larger diameter, or PDL coated fibers, respectively. For comparison of artificial axon stiffness and diameter, wrapping was defined as engagement of MBP+ processes or membrane around more than 80% of the pillar circumference and spanning any length of the pillar, as quantified by confocal microscopy and customized image analysis (**A**,**C** arrowheads). For comparison of artificial axon coatings, wrapping was defined as concentric coverage of >80% MBP+ rings of segment length >30 µm along the fiber (**E**, arrowheads). Wrapping occurred on a higher percentage of artificial axons that were stiffer (140 kPa vs. 0.4 kPa), thinner (10 μm vs. 20 μm), and laminin-coated (laminin vs. PDL). Experimental details summarized in Supplementary Table S2. All relative differences were reproducible across two independent trials, with no statistically significant differences between trials by two-tailed Fisher’s exact test (Supplementary Table S3).

The capacity to manipulate independently the multiple features of individual artificial axon fibers, fiber arrays, and the surrounding environment enables systematic interrogation of individual cues on oligodendrocyte response. These physical and biochemical cues can be engineered to represent specific components of the nervous system or disease environments. To demonstrate this capacity, we varied fiber diameter (Fig. 5A,B), stiffness of the fiber material (Fig. 5D,E) and surface coating (Fig. 5G,H) for vertically and horizontally oriented artificial axon arrays fabricated with poly(HDDA-*co-*starPEG) by PµSL. Such feature variations may represent the increase of axon diameter upon swelling^46^, decreased local stiffness^32,33,47^ and ligand expression changes in the axon surface or extracellular matrix^48^. These variations in diameter, stiffness, and surface functionality were facilitated by our modification of digital masks, polymer precursor composition, or post-fabrication surface modification, respectively. We achieved pillar diameters of 10–20 µm, and separately probed two levels of mechanical stiffness spanning three orders of magnitude (*E* = 0.42 ± 0.14 and 140 ± 35 kPa), while maintaining pillar height of up to 70 µm. These features can also be varied as a function of position within an array, enabling design and fabrication of heterogeneous microenvironments with high precision; see Supplementary Fig. S4.

To compare the effect of fiber stiffness and diameter on myelination, we quantified the extent of oligodendrocyte engagement with artificial axons, defined herein as detection of MBP+ processes or membrane around more than 80% of the pillar circumference and spanning any length of the pillar, as quantified by confocal microscopy and customized image analysis (see Materials and Methods, Imaging, data acquisition and statistical analysis). This comparison indicated approximately threefold greater oligodendrocyte engagement with the stiffer (140 kPa) versus more compliant (0.4 kPa) artificial axons, for laminin-coated pillars exhibiting diameter ~16 μm. Figure 5G represents this comparison as quantified by the fold increase as a function of artificial axon stiffness, determined from the percentage of MBP-engaged pillars in each array in each of two independent experiments (n > 35 pillars analyzed in each array). This finding was consistent with our previous studies of oligodendrocyte differentiation on flat hydrogels spanning this stiffness range^24^, and supports the hypothesis that pathological changes in local stiffness could alter myelination potential of oligodendrocytes via established mechanotransductive signaling networks^31^.

We also observed approximately threefold greater engagement by oligodendrocytes on artificial axons of the smaller (10 µm) versus larger (20 µm) diameter, for laminin-coated pillars exhibiting axon-like stiffness of 0.4 kPa^14^. Figure 5G illustrates this comparison as fold increase in the percentage of wrapped artificial axons, determined from the percentage of myelin-engaged fibers in each array for each of two independent experiments (n > 280 pillars analyzed in each array). Finally, we observed approximately threefold greater myelination on artificial axons coated with a common ligand relevant to oligodendrocyte biology (laminin) as compared to a surface coating promoting nonspecific cell adhesion (PDL), Fig. 5G. Here, we compared the percentage of horizontal fibers exhibiting full wrapping, defined herein as concentric coverage of >80% MBP+ rings of segment length >30 µm along the fiber in each of two independent experiments (n > 300 fibers analyzed in each array). On average, 36% of all laminin-coated fibers exhibited full wrapping, as compared to 12% of PDL-coated fibers (see Supplementary Table S3), at constant fiber diameter (10 µm) and stiffness (140 kPa).

For each of these three comparisons of cell response as a function of artificial axon stiffness, diameter, or surface coating in Fig. 5, relative differences were reproducible across two independent trials, with no statistically significant differences between trials by two-tailed Fisher’s exact test (Supplementary Table S3).

## Discussion

We demonstrated the design, fabrication and implementation of artificial axon arrays that enable direct observation of oligodendrocyte responses in an *in* vitro environment mimicking biological axons. Using recent advances in additive manufacturing and materials processing, we developed the first such arrays with fibers that are mechanically compliant (0.1–1000 kPa), aligned, minimally supported, and of small (5–20 µm) diameter (Fig. 3). Although multiple cues modulate cell behavior, the capacity to create such uniformly cylindrical axon-like fiber arrays with mechanical stiffness approaching that of neuronal axons addresses the emerging need to understand and replicate physical cues of the glial cell microenvironment, which vary with disease state and can affect cell differentiation and myelination. This approach provides artificial axons with stiffness within the order of magnitude of biological axons^14^, and up to six orders of magnitude more compliant than state-of-the-art materials currently used for myelination assays (e.g., glass, polystyrene, polylactic acid and polycaprolactone)^5,9–11,49,50^.

The materials used herein to print fibers as artificial axons (PDMS, polyHEMA and poly(HDDA-*co-*starPEG)) are amenable to surface functionalization with ligands that can be chosen to reflect particular healthy or pathological microenvironments. We demonstrated that OPCs adhere to, engage and migrate along fibers and across fiber bundles (Fig. 4 and Supplementary Fig. S2) coated with poly-D-lysine (used here as a non-specific binding control, providing electrostatically driven cell attachment without integrin-dependent signaling) or laminin. Laminin is expressed on the axon surface, and has been shown to regulate myelination via integrin-dependent pathways; oligodendrocytes express laminin-binding integrins^49^. OPCs on these functionalized artificial axons differentiated into MBP+ oligodendrocytes that ensheathed fibers with up to 120 µm-long segments wrapped around the entire fiber circumference (Fig. 4 and Supplementary Fig. S2). The versatility of these printing methods and materials allowed us to manipulate physical, biochemical and mechanical properties of artificial axons with high control and precision, to reflect different cues characteristic of disease environments such as demyelinating lesions (Fig. 5).

Disease microenvironments often present complex structure of biochemical and biophysical features, the spatial arrangements of which may be important factors in a disease. For example, tumor or demyelinating lesion environments demonstrate spatial gradients of stiffness^33,47^ and acidity^51,52^ as well as changes in cellular composition and molecular components of extracellular matrix^48^. We and others have demonstrated that these biochemical and biophysical phenomena confer significant effects on oligodendrocyte lineage cells *in vitro*, affecting survival, proliferation, migration and differentiation^24,27,31,52^ which are also critical processes *in vivo*. However, effects of these cues on actual process of myelination have been impossible or very difficult to study systematically *in vitro*. Prior methods have taken advantage of the geometrical scales achievable by electrospinning^8,9,50^. While single-ligand and axon diameter variation have been studied as cues for myelin ensheathment in electrospun fiber platforms, the range of biophysical and biochemical cues that may be investigated are limited currently by the capabilities of this manufacturing modality. Mechanical properties of electrospun fibers are restricted to supraphysiological magnitudes (i.e., high stiffness) due to the materials optimized thus far by the electrospinning community. The present study contributes to the growing evidence of glial cell mechanosensitivity^23–31^ and demonstrates the first evidence that initiation of myelin ensheathment may be modulated by axon stiffness when other cues are maintained invariant (Fig. 5). We postulate that matching the mechanical properties of biological axons in a reductionist *in vitro* myelination model will aid more accurate prediction of oligodendrocyte response to pro-myelinating agents *in vivo*. The tools developed in this study will be useful for future studies that evaluate whether compaction and extent of myelin ensheathment observed *in vivo* may be recapitulated and modulated in a mechanically matched axon-free model.

Another important consideration of such engineered platforms is the reproducibility and fine control of relative position and physical features of artificial axons. Such control may be required to address biological questions about the axon-glia complex, e.g., the effect of increased interaxonal spacing on peripheral nerve remyelination within a demyelinating lesion^53^. Electrospinning can provide coarse alignment via rotating collectors^8,9^ with limited reproducibility of alignment and spacing, generally restricted to multiple layers of fibers^8,9,13,54,55^. Moreover, the parameters of that dynamic fiber collection process are coupled directly to other spatial parameters (e.g., fiber spacing and diameter) and material properties of the fibers and the nonwoven mat (e.g., porosity and stiffness)^55^. That coupling can obfuscate direct comparison between experimental conditions of interest. An additional degree of spatial control may be achieved with modified collector geometries^55,56^, but require additional resources and complicate experimental design, fabrication and sample handling. In our PµSL-based approach, spatial parameters are manipulated through digital masks (Fig. 2) and significantly decoupled from process and material parameters, enabling generation of a large number of samples with any number of predetermined variations (Fig. 5 and Supplementary Fig. S4) with relative ease, speed and precision.

Precise fiber alignment is desirable not only to mimic the physiological orientation of axon bundles in the nervous system, but also to facilitate image acquisition and expedite image-based quantification of myelin ensheathment. Mei *et al*. capitalized on the precision and reproducibility of photolithography patterning to develop a reductionist high-throughput myelination assay, and describe these as micropillar arrays^13^. In contrast to electrospun fiber platforms, Mei *et al*.’s platform facilitated automated MBP+ membrane identification and quantification with a rational design that aimed to speed visual identification of myelination for screening of drug candidates. A key limitation of that important approach was the compromising of biological fidelity in terms of several parameters: shape (shallow cone versus a cylindrical axon of high aspect ratio), dimensionality (diameter gradient along cone height versus an axon’s relatively uniform diameter over that span) and mechanical properties (fused silica *E* ~ 10^10^ Pa versus an axon’s *E* ~ 10^2^–10^3^ Pa). Furthermore, while the sparse distribution of glass cones in that array facilitated data acquisition, it also limited interaction of OPCs with multiple cones or cells as could occur in axon bundles; those interactions have been proposed to impact OPC differentiation and myelination *in vitro* ^7^. In our platforms of mechanically compliant artificial axons, cylindrical features were arranged in close proximity (Figs 1 and 4), with OLs often surrounded by and interacting with at least two artificial axons (Fig. 4 and Supplementary Fig. S2). This interaxonal distance can also be varied with high precision.

Here we addressed the effect of artificial axon stiffness on oligodendrocyte engagement and myelination, as well as the effects of diameter and surface ligand in aligned arrays. We showed that significantly more artificial axons were ensheathed by oligodendrocytes engaging laminin-coated vs. poly-D-lysine coated artificial axons (Fig. 5G). This result was consistent with previous findings for oligodendrocytes grown on much stiffer (~1 GPa) electrospun fibers of ~1 µm diameter^9^, and earlier studies that demonstrated the stimulating role of laminin in axon myelination^49,57^. However, our approach afforded the comparison of ligand type or density on fibers of physiological stiffness (<1 kPa). Furthermore, the precise alignment enabled by 3D printing afforded rapid image-based quantification for reliable identification of full circumferential wrapping (and live cell imaging; Supplementary Movies S1–S5) by confocal microscopy. Artificial axon diameter also affected oligodendrocyte engagement, with OL processes and MBP+ membranes more likely to engage, or at least partially wrap, pillars of 10 µm diameter than 20 µm diameter (Fig. 5G). The infrequent observation of oligodendrocyte engagement with artificial axons of 20 µm diameter could indicate that there exists a maximum permissive diameter threshold above which full myelin-rich wrapping may not proceed efficiently. Of course, a wider range of diameters must be considered to explore this point further, while maintaining mechanical and biochemical features invariant.

Further, oligodendrocytes were approximately three times more likely to engage artificial axons of 140 kPa stiffness, as compared to more compliant counterparts approximating the sub-kPa stiffness of biological axons (0.4 kPa^14,15^) (Fig. 5G). Here, we demonstrate chiefly that material stiffness spanning the range 0.4 kPa to 140 kPa can modulate the process of myelin wrapping in a 3D axon-like environment. However, as discussed in Supplementary Information (brief discussion of prior results for flat polymers and CNS microenvironment stiffness), we did not intend this as a generalized claim that myelination is always promoted on stiffer fibers; these fibers were of a specific diameter (~16 μm) and ligand coating (laminin). Instead, we note that the mechanically matched artificial axon materials developed herein enable studies of myelination within the low stiffness range of axons in the CNS (<10^3^ Pa)^14^, thus providing a more accurate mechanical environment than previous models based on polymers or glass that are stiffer by several orders of magnitude (10^5^–10^10^ Pa).

Overall, these observations indicate plausibly that changes occurring in the axon physical and surface chemical properties can affect oligodendrocyte repair of myelination in biological axons, a hypothesis that deserves future study and can be aided by such platforms. The demyelinating lesion environment includes reported changes in ECM composition^47,48^, tissue stiffness^32,33,47,58,59^ and axon swelling^46^; our earlier^24,31^ and current findings predict that such changes can in turn alter oligodendrocyte differentiation and potential to repair myelin. The axon stiffness, diameter, and ligand presentation can each play a role in oligodendrocyte response. The effect of these cues may or may not be coupled, and this study was not designed to compare the relative or coupled contributions of such physical properties in oligodendrocyte responses such as extent of wrapping. However, future studies can now continue to explore the role of axon-like mechanical stiffness in the process of myelination for even smaller fiber diameters of ~1 µm, given the current understanding that axon diameter in this range can act as a permissive or restrictive cue for oligodendrocyte engagement and wrapping of axons^8^. Further interrogation of the engaged and wrapped artificial axons to document myelin sheath structure is also desirable, although our goal in this study was to demonstrate MBP-rich engagement of the printed fibers by oligodendrocytes, for rapid optical imaging of the intact and hydrated structures. Functional myelination *in vivo* is described more completely by multilayered compaction of myelin, and typically imaged destructively through transmission electron microscopy. While we observed through confocal microscopy that MBP+ segments of >30 μm length span surrounded the entire circumference of artificial axons, it remains to be determined whether these segments recapitulate the compact multilayered sheath structure. It will be useful to determine whether the extent of MBP coverage as assessed by confocal microscopy can be a correlative marker for compact myelination in this system. This would be particularly beneficial for application of this technology in high throughput screening that does not require sample drying and extensive sample preparation. Future approaches for analysis compatible with this material platform can include the standard transmission electron microscopy, or cryogenic focused ion beam scanning electron microscopy and x-ray microtomography to facilitate three-dimensional image acquisition and analysis of large arrays for such correlative analysis of rapid optical imaging.

While the present study focused on axon biophysical properties, a myriad of cellular and extracellular components may contribute to the formation of a functional myelin sheath. Optimizing culture conditions, including soluble media factors and artificial axon surface chemistry, and incorporating additional components of the extracellular matrix, may improve the quality of myelination and throughput of myelination assays in this platform. Indeed, the materials and methods discussed herein are amenable to a wide range of surface, bulk and soluble chemistries, and are not limited to the functional ligands, adhesion molecules and media used in this particular study.

By harnessing this toolbox of artificial axon arrays and associated image analysis, we can now create engineered environments that reflect key physiological (and pathological) mechanical, geometric, and biochemical components of the glial microenvironment for myelination assays. More generally, this approach enables synthesis of diverse and disease-representative microenvironments with higher fidelity and more reproducibly than existing platforms. Such advances offer new potential for high throughput analyses of cell response to physical cues or drug responsivity, and ultimately for predictions of *in vivo* outcomes.

## Materials and Methods

### Ethics Statement

This study was carried out in accordance with the guidelines of the National Institutes of Health for animal care and use (Guide for the Care and Use of Laboratory Animals) and the protocol was approved by the Institutional Animal Care and Use Committee at the Massachusetts Institute of Technology (MIT Committee on Animal Care).

### Purification and culture of rat oligodendrocyte precursor cells

OPCs were isolated from Sprague Dawley rat (BioreclamationIVT) mixed glial cultures, as described previously^24^. Briefly, mixed glial cultures obtained from neonatal cultures were maintained for 10–14 days in 10% fetal bovine serum (FBS, Atlanta Biologicals) and DMEM (Gibco) and shaken overnight at 37 °C and 5% CO_2_ to detach OPCs. After shake-off, OPCs were purified from microglia in P60 dishes by differential adhesion to untreated polystyrene. OPCs were maintained in progenitor state in DMEM with SATO modification (5 mg/mL insulin, 50 mg/mL holo-Transferrin, 5 ng/mL sodium selenate, 16.1 mg/mL putrescine, 62 ng/mL progesterone, and 0.1 mg/mL bovine serum albumin), 10 ng/mL platelet-derived growth factor homodimer AA (PDGF-AA, Peprotech) and 10 ng/mL basic fibroblast growth factor-2 (FGF-2, Peprotech) (proliferation medium). Differentiation was induced after 24 h–48 h in SATO’s medium with 0.5% FBS, without PDGF-AA and FGF-2 (differentiation medium).

### Fabrication of PDMS fibers

#### Substrate

PDMS fibers were first printed on a layer of smooth aluminum foil, and then transferred onto glass slides or custom-made PDMS plates (Supplementary Fig. S5). *Resin*: PDMS ink was made using SE1700 (Dow Corning), 10:1 w/w base to hardener ratio, and 0.01% w/w Rhodamine B as a fluorescent marker for fiber imaging. Components were mixed in a centrifugal deaerating mixer (Thinky Mixer) for 6 min, then loaded into the syringe, spun for 10 min in the centrifuge for degassing, and used immediately. *Fabrication*: Direct writing requires specific rheological characteristics of the extruded material: polymer “inks” that are yield stress fluids. Specifically, these inks must be tailored to facilitate flow through the deposition nozzle under an applied shear stress, yet retain its filamentary shape upon exiting the nozzle^43^. A syringe with degassed ink was mounted on a custom 3D printing setup; the ink was extruded through a glass nozzle with 10 µm inner diameter. The top layer of fibers was first printed on the smooth aluminum foil and pre-cured for 30 min at 80 °C, followed by printing supporting beams, sandwiching with either a glass slide or PDMS custom plate, and curing at 80 °C for 2 h. After curing, the top layer of aluminum was gently removed, leaving behind the undisturbed top layer of fibers (Supplementary Fig. S5). This “top-to-bottom” printing technique with pre-curing step allowed us to produce overhanging fibers. If printed directly on the support beam, PDMS fibers with diameters as small as 10 µm sag and collapse to the substrate surface before curing.

### Fabrication of poly-HEMA fibers

#### Substrate

Poly-HEMA fibers were printed on clean glass slides. *Resin:* The formulation of pHEMA inks is described elsewhere in detail^41^. Briefly pHEMA inks with varying concentrations of high molecular weight pHEMA chains (1 MDa and 300 kDa, Sigma-Aldrich), HEMA monomer (Sigma-Aldrich), ethylene glycol dimethacrylate (EGDMA) comonomer (Polysciences), Irgacure 2959 photoinitiator (BASF), ethanol, and deionized water were prepared (Supplementary Fig. S6). Each ink was produced by first combining all the liquid components with Irgacure until it was dissolved using brief (15 s) sonication; the solid components were then added (high molecular weight pHEMA). The mixture was placed in a 20 mL centrifugal mixing container (Thinky mixer), mixed at 2000 rpm for 5 min, and left to sit in a light-free container for 72 h at 4 °C where the pHEMA chains relax and disperse in the solvent to create a highly viscous ink. Finally, the pHEMA ink was loaded into a UV-proof syringe and spun in the centrifuge for degassing. The ink can be used immediately or stored protected from light in 4 °C for up to 6 weeks. *Fabrication:* pHEMA fibers were printed as multi-layer logpile constructs directly on glass slides using a tapered glass nozzle with 10 µm inner diameter. The fibers maintain their suspended shape before curing allowing for printing of multiple fiber layers without a pre-curing step. Printed constructs were then UV-cured using an Omnicure UV lamp, and stamped to the glass substrate with a rim of PDMS, which was next thermally cured. The cured constructs were washed in sterile water for 7 days, before functionalization for cell culture.

### Fabrication of poly(HDDA-co-starPEG) fibers

#### Substrate

Poly(HDDA-*co-*starPEG) fibers were fabricated on 12-mm coverslips functionalized as previously described^38^. Coverslips were rinsed with acetone and ethanol to remove impurities, blown dry with air, and exposed to air plasma for 5 minutes. Activated coverslips were functionalized with 2% v/v 3-(Trimethoxysilyl)propyl methacrylate (Sigma-Aldrich) and 0.01% v/v acetic acid in ethanol at room temperature for 2 h, to introduce acrylate groups on the surface that bind to the photopolymerized structures during PµSL fabrication. Coverslips were subsequently rinsed twice with ethanol, blown dry, and stored in a desiccator for up to 6 months. *Resin:* This method requires resins that are liquid at room temperature, of low viscosity, and that can cure quickly and locally under UV. Poly(HDDA-*co*-starPEG)-high-E resin was prepared by mixing 10% w/w 4-arm PEG acrylate (starPEG, 20 kDa arms, Creative PEGWorks), 30% w/w HDDA (Sigma), 2% w/w Irgacure 819 (phenylbis(2,4,6-trimethylbenzoyl)phosphine oxide, Sigma), 0.7% w/w Sudan I (Sigma) and 0.1% w/w Rhodamine B (Sigma) in DMSO, and sonicating at 37 °C for 10 min. Poly(HDDA-*co*-starPEG)-low-E resin was prepared similarly, with 10% w/w 4-arm PEG acrylate and 10% w/w HDDA. Resins were stored in opaque containers at room temperature for up to 1 month. *Fabrication:* Fabrication of fibers and pillars was enabled by the PµSL apparatus specifically tailored for tissue engineering^60^. A CAD model was sliced to obtain cross-sectional images of the 3D structure at different heights as digital masks. These masks were sent to a 1920×1080 resolution, 5 µm pixel-size, TI (Texas Instruments) manufactured DMD (Digital Micromirror Device) chip taken from a commercial projector (Acer H6500). The chip was illuminated by a light source purchased from Hamamatsu with high intensity and peak wavelength around 365 nm. Between the DMD chip and the fabrication plane, a 10:1 composite lens from Carl-Zeiss with resolution of 1 µm was used to project image onto the resin surface to be cured. Each image exposed at the print-plane immediately solidified a layer at the top of the resin bath – the thickness of each layer is determined by the light penetration depth in the resin and the vertical step size of a three linear motion stage from Aerotech. Light intensity and exposure time determine the crosslinking density of the polymer, allowing for variation in elastic modulus, viscosity, permeability, and swelling ratio. The cured layer was then lowered to print the next layer. The process was repeated and the entire CAD model was fabricated in a layer-by-layer manner. In stereolithography, controlled oxygen inhibition above the cured sample surface is important for fast print speed while retaining high feature resolution^59,61^. An oxygen permeable PDMS window was placed above the UV projection plane to maintain a thin layer of uncured resin between the window and the cured sample throughout fabrication, as previously described^60^. Separation forces between the window and the cured sample can be very large and destructive to the sample. We found that part separation became increasingly problematic in PµSL with increased material hydrophilicity and low mechanical stiffness, and separation forces remained large even with the use PDMS windows. Alternative coatings to PDMS such as fluoropolymers reduced the magnitude of separation forces, and prevented absorption of resin components, which significantly improved print quality and throughput. A stitching operation was performed by controlling the stage motions in the XY direction to provide a large build size without compromising XY resolution. Horizontal fiber modules consisted of 6 10 µm-thick support beam layers and one fiber layer of 10 µm-thickness, with exposure of 1.85 s/layer (poly(HDDA-*co*-starPEG)-high-E) or 2.6 s/layer (poly(HDDA-*co-*starPEG)-low-E). Vertical fibers or pillars consisted of seven layers each of 10 µm-thickness, with exposure of 1.85 s/layer (poly(HDDA-*co*-starPEG)-high-E) or 2.6 s/layer (poly(HDDA-*co-*starPEG)-low-E). Fibers were washed overnight in 100% ethanol, followed by at least 48 h in PBS. Washed fibers were sterilized under UV for 10 min inside the biosafety cabinet, rinsed once with sterile PBS, and stored for up to a month in PBS at 4 °C prior to functionalization. Fluorescent signal from dyes incorporated within fibers was strong for at least 1 month stored in PBS. Rhodamine B introduced noise in both green and blue channels of available confocal microscopy fluorescent filters, but remained below the signal of MBP and Hoechst stains used to identify myelin and oligodendrocyte cells; however, other dyes may be used.

Although fiber feature resolution of PµSL is limited to 0.5–1 µm, which covers a wide range of diameters in both CNS and PNS in humans, two-photon stereolithography is an attractive alternative to fabricate sub-µm artificial axons, using the same materials and similar approach. The quadratic dependence of two-photon absorption confines the photopolymerization to nano-volumes, which would allow for artificial axons with more physiological dimensions found in the human CNS. However, this translates to very slow and expensive printing, which currently precludes scale-up for commercial or high-throughput applications.

### Artificial axon functionalization

Before cell seeding, fibers were functionalized with one of three ligands: poly-D-lysine (PDL MW 70,000, Sigma), laminin (mouse natural laminin from Engelbreth-Holm-Swarm (EHS) sarcoma, Invitrogen), or fibronectin (from bovine plasma, Sigma). *PDMS fibers:* Fibers were washed in acetone (12 h) followed by wash in ethanol (12 h), to remove uncured species. After drying in the oven at 45 °C overnight, the PDMS fibers were activated in air plasma for 20 min to render them hydrophilic, followed by incubation with 100 mM (3-Aminopropyl)triethoxysilane (APTES, Sigma) at room temperature to introduce NH_2_ groups to the silicone surface, and washed three times with deionized water. The fibers were incubated for 4 h at room temperature with a mixture containing 1 mM solution of bis(sulfosuccinimidyl)suberate crosslinker (BS3, Covachem) and 100 µg/mL solution of ligand (fibronectin, laminin, or PDL) in HEPES buffer (50 mM, pH 8.0) to facilitate chemical attachment of the ligand to NH_2_ groups on fiber surface, followed by three washes with 1x phosphate buffer saline (PBS, pH 7.4). *pHEMA fibers:* Fibers were incubated overnight with 100 µg/mL solution of ligand (fibronectin, laminin, or PDL) in 1x PBS. *Poly*(*HDDA-co-starPEG*) *fibers:* Washed and sterilized fibers were incubated overnight in 50 µg/mL solution of ligand (laminin or PDL) in 1x PBS, and subsequently washed three times with 1x PBS. Coverslips were immobilized in 6-well plates using either high vacuum grease (Dow Corning) or a hydrophobic barrier pen (PAP pen, Vector Labs). For all printed materials, the efficiency of ligand deposition was verified with 50 µg/mL fluorescently labeled poly-L-lysine (poly-L-lysine-FITC MW 15–30 kDa, Sigma) and laminin (Laminin-rhodamine, MW 225–400 kDa, Cytoskeleton). Following functionalization, all fibers were washed once with SATO’s medium and incubated for at least 1 h in proliferation medium before seeding. OPCs were seeded at densities of ~50,000 cells/cm^2^.

### AFM-enabled nanoindentation and rheology

The rheological properties of the pHEMA inks were determined using a controlled stress rheometer (DHR-3, TA Instruments, New Castle, DE, USA) fitted with a cone and plate geometry with a 40 mm diameter, 2° cone. Shear viscosity measurements were carried out in controlled shear stress (τ) mode in a logarithmically ascending series of discrete steps. The elastic shear (*G*′) and viscous (*G*′′) moduli were measured using an oscillatory logarithmic stress sweep at a frequency of 1 Hz. Measurements were carried out at 22 °C using an aqueous solvent trap to mitigate drying effects.

The Young’s elastic modulus *E* was determined for fibers manufactured by both methods with all materials studied. Thin films of each material (10 µm thickness and width) were fabricated by direct printing and PµSL using the same parameters as for artificial axons, and equilibrated overnight in PBS. Atomic force microscope (AFM)-enabled nanoindentation measurements were conducted (MFP-3D Bio, Asylum Research) using cantilevers of nominal spring constant *k* = 0.03 N/m and 0.3 N/m, terminating in a borosilicate spherical probe (Novascan) with an approximate diameter of 2 µm. The actual spring constant was calibrated as previously described^42^. Between 10 and 20 force-depth responses were collected from one sample of each material, in PBS. For the most compliant materials, the cantilever base velocity was 1 µm/s and probe retraction was triggered after reaching a maximum force of 0.2 nN. For the stiffer materials (*E* > 100 kPa) the cantilever base velocity was 1 µm/s and probe retraction was triggered after reaching a maximum force of 30–100 nN. Young’s elastic moduli *E* were calculated by fitting the spherical Hertz model, as previously described^24^, to a depth of 200 nm, or approximately 10% strain, and reported as ±s.e.m.

### Immunocytochemistry

Cells were fixed with 4% paraformaldehyde, washed with PBS, permeabilized with 0.1% Triton X-100 for 5 min, and blocked with 1% bovine serum albumin in PBS and 0.1% Triton-100 (blocking solution) for 1 h. Primary antibodies (rat anti-MBP, 1:200 dilution, Serotec) were diluted in blocking solution and incubated at room temperature for 1 h. Samples were washed 3 times with PBS and incubated with secondary antibodies (rabbit anti-rat IgG Alexa Fluor 488, 1:200 dilutions, Invitrogen) in PBS for 1 h, followed by washing and staining of nuclei with Hoechst 33342 at a 1:1000 dilution for 5 min.

### Imaging, data acquisition and statistical analysis

Phase contrast images were acquired with an inverted microscope (Olympus IX-81) equipped with an Orca-R2 camera. Fiber z-stacks were acquired with an inverted laser scanning confocal microscope (Olympus FV1000). Three-dimensional volumes were reconstructed from z-stacks using Fiji 3D Viewer; analysis of myelin segments and wrapping was done using the Volume Viewer plugin. The percentage of engaged pillars (Fig. 5G, stiffness comparison and diameter comparison) was defined as the number of pillars wrapped around more than 80% of the pillar circumference by MBP+ processes or membrane spanning any extent of the pillar length. In Fig. 5G, myelin-engaged pillar fraction of 140 kPa array was normalized by that of 0.4 kPa array in each of two independent experiments, with 36 to 85 pillars analyzed in each array (See pillar diameter distributions in Supplementary Fig. S3. For stiffness comparison, only pillars within 6% of the targeted printed diameter were included in subsequent cell response analysis, resulting in a smaller subset of analyzed pillars within each array as compared to the arrays of varied stiffness or ligand coating.) Myelin-engaged pillar fraction of the 10 μm-diameter pillars was normalized by that of the 20 μm-diameter pillars in each of two independent experiments, with 335 to 552 pillars analyzed in each array (see pillar diameter distributions in Supplementary Fig. S3). The percentage of fully wrapped fibers (Fig. 5G, coating comparison) was defined as the number of fibers wrapped around more than 80% of the fiber circumference, and presenting MBP+ segments extending longer than 30 µm along both the top and bottom of the fiber length. In Fig. 5G, fully wrapped fiber fraction in the laminin-functionalized fiber array was normalized by that determined for the PDL-functionalized fiber array in each of two independent experiments, with 317–408 fibers analyzed in each array. Statistical analysis was performed using two-tailed Fisher’s exact test (Supplementary Table S3) to determine the reproducibility of the observed fold-increase or relative difference in the percentage of wrapped artificial axons between two conditions (e.g., laminin vs. PDL) for n = 2 independent experimental trials, and expressed as *p < 0.05.

### Data availability

The authors declare that the data supporting the findings of this study are available within the paper and its supplementary information files. Further relevant data are available from corresponding authors on request.

## Electronic supplementary material

Supplementary Information

Supplementary Movie S1

Supplementary Movie S2

Supplementary Movie S3

Supplementary Movie S4

Supplementary Movie S5
