# Supplementary material for: Engineered 3D-printed artificial axons
Source: Sci Rep. 2018 Jan 11;8:478. doi: 10.1038/s41598-017-18744-6 (PMC5765144; doi:10.1038/s41598-017-18744-6)
Supplement: Supplementary file 1 — Supplementary Information [file 41598_2017_18744_MOESM1_ESM.pdf]

Engineered 3D-printed artificial axons

Daniela Espinosa-Hoyos, Anna Jagielska, Kimberly A. Homan, Huifeng Du, Travis Busbee, Daniel G. Anderson, Nicholas X. Fang, Jennifer A. Lewis, Krystyn J. Van Vliet

Supplementary Information

Contents:

Table S1-S3
Figures S1-S7
Movies S1-S5
Brief discussion of prior results for flat polymers and CNS microenvironment stiffness
Reference 60

	PDMS	Poly-HEMA Low E	Poly-HEMA High E	Poly(HDDA-*co*-starPEG) 3:1	Poly(HDDA-*co*-starPEG) 1:1
Base	SE1700: 90% w/w base, 10% w/w hardener	10% pHEMA (1000 kDa) 25% pHEMA (300 kDa) 5% HEMA monomer 1% EGDMA (comonomer)	10% pHEMA (1000 kDa) 25% pHEMA (300 kDa) 40% HEMA monomer 1% EGDMA (comonomer)	30% w/w HDDA, 10% w/w starPEG	10% w/w HDDA, 10% w/w starPEG
Curing agent	Temperature: 80°C	0.3% w/w Irgacure	0.3% w/w Irgacure	2% w/w Irgacure 819	2% w/w Irgacure 819
Light absorber	-	-	-	0.7%, Sudan I	0.7%, Sudan I
Fluorescent dye	0.01%, Rhodamine B	0.01%, Rhodamine B	0.01%, Rhodamine B	0.1%, Rhodamine B	0.1%, Rhodamine B
Solvent	-	25% ethanol, 33.7% water	23.5% water	DMSO	DMSO

Table S1. Material composition and processing details

Experiment trial #	Orientation	Stiffness (kPa)	Diameter (μm)	Coating
1	Vertical	0.4	10	Laminin
2	Vertical	0.4	10	Laminin
1	Vertical	0.4	20	Laminin
2	Vertical	0.4	20	Laminin
**Experiment trial #**	**Orientation**	**Stiffness (kPa)**	**Diameter (μm)**	**Coating**
1	Horizontal	140	10	Laminin
2	Horizontal	140	10	Laminin
1	Horizontal	140	10	PDL
2	Horizontal	140	10	PDL
**Experiment trial #**	**Orientation**	**Stiffness (kPa)**	**Diameter (μm)**	**Coating**
1	Vertical	0.4	16	Laminin
2	Vertical	0.4	16	Laminin
1	Vertical	140	16	Laminin
2	Vertical	140	16	Laminin

Table S2. Experimental details for arrays represented in Fig. 5.

Stiffness (% Wrapped pillars)			
	**0.4 kPa**	**140 kPa**	**140 kPa/0.4 kPa**
Trial 1	1.18%	3.75%	3.19
Trial 2	2.78%	6.98%	2.51
		**p-value**	1
**Diameter (% Wrapped pillars)**			
	**10 μm**	**20 μm**	**10 μm/20 μm**
Trial 1	9.60%	3.58%	2.68
Trial 2	4.92%	1.71%	2.87
		**p-value**	1
**Coating (% Wrapped fibers)**			
	**Laminin**	**PDL**	**Laminin/PDL**
Trial 1	8.40%	32.51%	3.87
Trial 2	15.77%	40.20%	2.55
		**p-value**	0.3489

Table S3. Contingency tables and p-values from two-tailed Fischer's exact test on the fold-increase in the percentage of wrapped artificial axons for two independent experiments (trial 1 and 2) that explored the same parameter comparison (two stiffnesses, two diameters, or two coatings).

The two-tailed Fisher's exact test was used to consider any significant differences in the fold increase in the percentage of wrapped pillars (rightmost column) between two independent experimental trials using different 3D-printed arrays and preparations of cells. No significant difference was identified between these two trials, confirming repeatability of the observed fold-

increase for a given fiber property that was varied while retaining the other two properties constant (stiffness, diameter, or coating).

Note that there is no experimental uncertainty associated with the percentage of wrapped pillars in a given trial or given condition (e.g., Trial 1, stiffness of 0.4 kPa) because the magnitude reported (e.g., 1.18% for that case) is the percentage of all the pillars imaged in a given array of pillars.

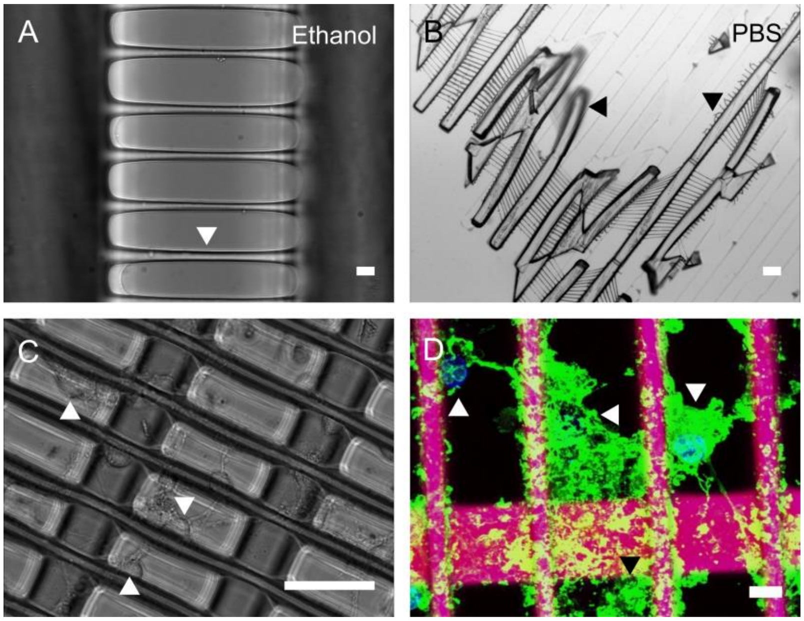

Figure S1. HDDA fibers, fiber failure and myelination. Hexanediol diacrylate (HDDA) is used commonly in stereolithography-based additive manufacturing to yield fine three-dimensional structures with mechanical stiffness that is approximately two orders of magnitude below that of polystyrene and glass. (A) HDDA fibers with diameters below 10 µm (arrowhead, 7 µm) can be manufactured with PµSL and are stable in organic solvents (e.g. ethanol, acetone, isopropanol, DMSO). Scale bar is 10 µm. (B) HDDA fibers are incompatible with water, saline solutions (PBS) and biological medium in concentrations above 25-50% v/v in ethanol, likely due to the high hydrophobicity of this material and surface tension phenomena. Silane coupling between HDDA microstructures and glass is inefficient. Fibers, supports and base layers break, and peel or lift from the underlying functionalized glass substrate. Scale bar is 100 µm. (C) Contrary to the incompatibility of OPCs with macroscopic (or bulk) HDDA substrates[42], OPCs have better survival on HDDA microfibers, differentiate and engage with HDDA fibers extensively. Scale bar is 100 µm. (D) Compressed z-stack of HDDA fibers myelinated by mature oligodendrocytes. Scale bar is 10 µm.

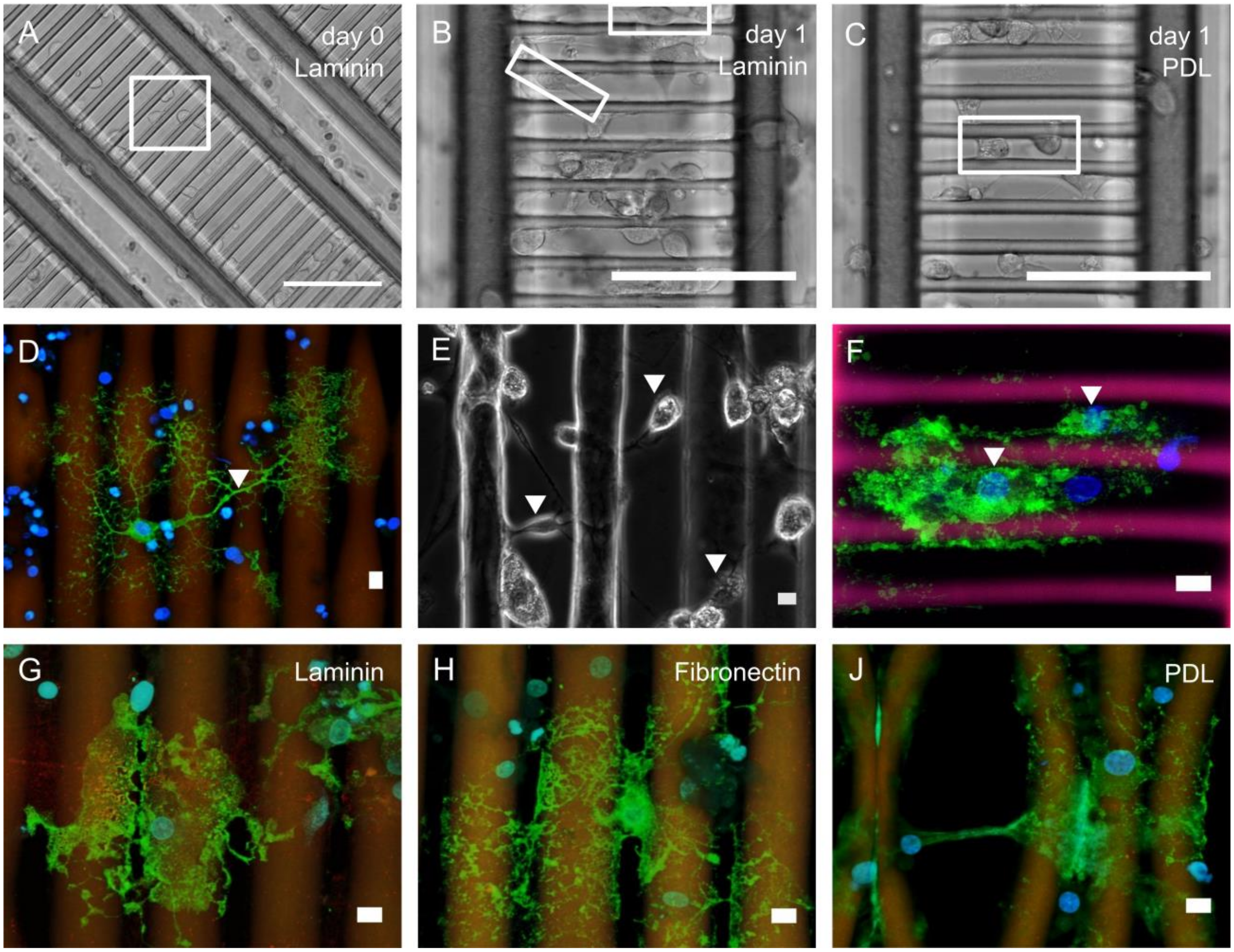

Figure S2. Cell behavior on artificial axons. (A) Cells adhere to poly(HDDA-*co*-starPEG) laminin-coated fibers within 1 hour with flattened morphology. (B) OPCs display bipolar morphology within 1 day in proliferation medium on laminin-coated poly(HDDA-*co*-starPEG fibers). (C) Fewer OPCs exhibit bipolar morphology and rather lack processes on poly(HDDA-*co*-starPEG) fibers coated with a nonspecific ligand (poly-D-lysine) within the same timeframe. (D) Some cells extended processes to fibers located up to 120 µm from the cell body (PDMS, fibronectin). (E-F) Oligodendrocyte somas often span the space between parallel fibers myelinating multiple fibers; (E) – pHEMA, laminin; (F) - poly(HDDA-*co*-starPEG, laminin. (G) On laminin-coated PDMS fibers, there is greater occurrence of membranous cells and fibers rather than highly branched cells. In contrast to laminin-coated PDMS fibers, there is higher occurrence of long oligodendrocyte branches rather than membranous fibers on (H) fibronectin and (I) PDL coated PDMS artificial axons. Scale bars are 100 µm in (A-C); scale bars are 10 µm in (D-J).

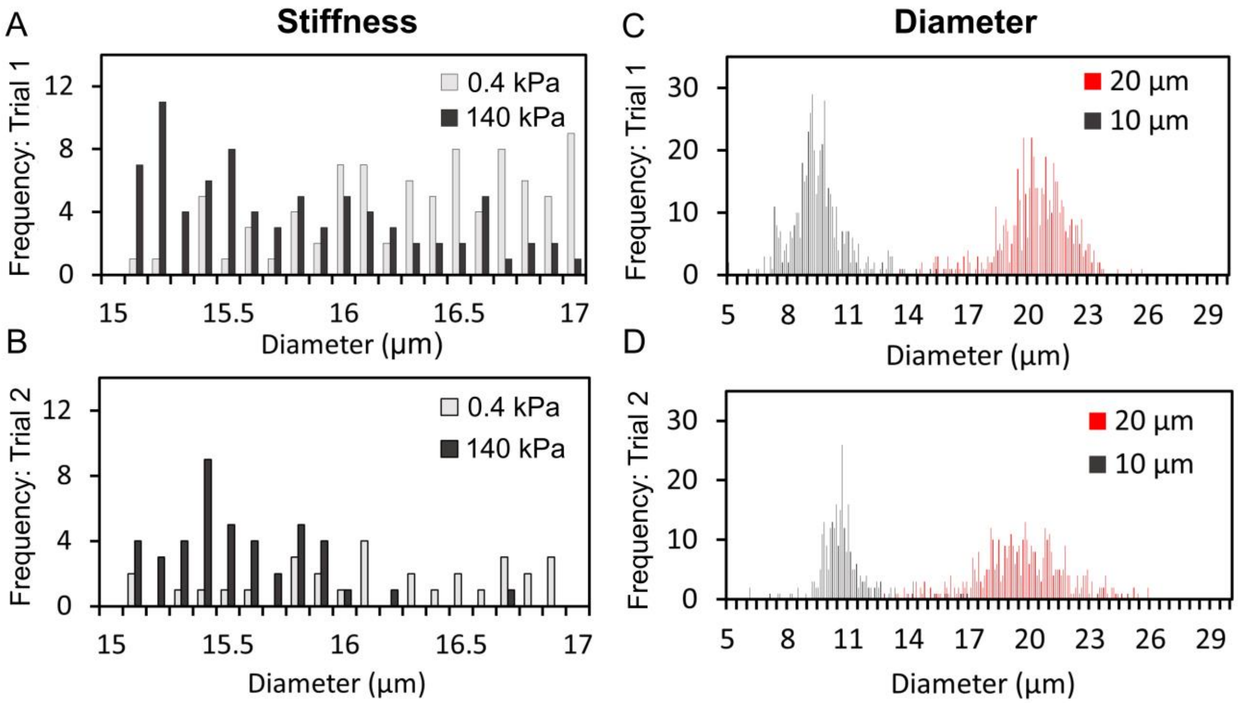

Figure S3. Distributions of pillar diameters for assessment of oligodendrocyte engagement with (A-B) compliant versus stiff, and (D-E) thick versus thin pillars.

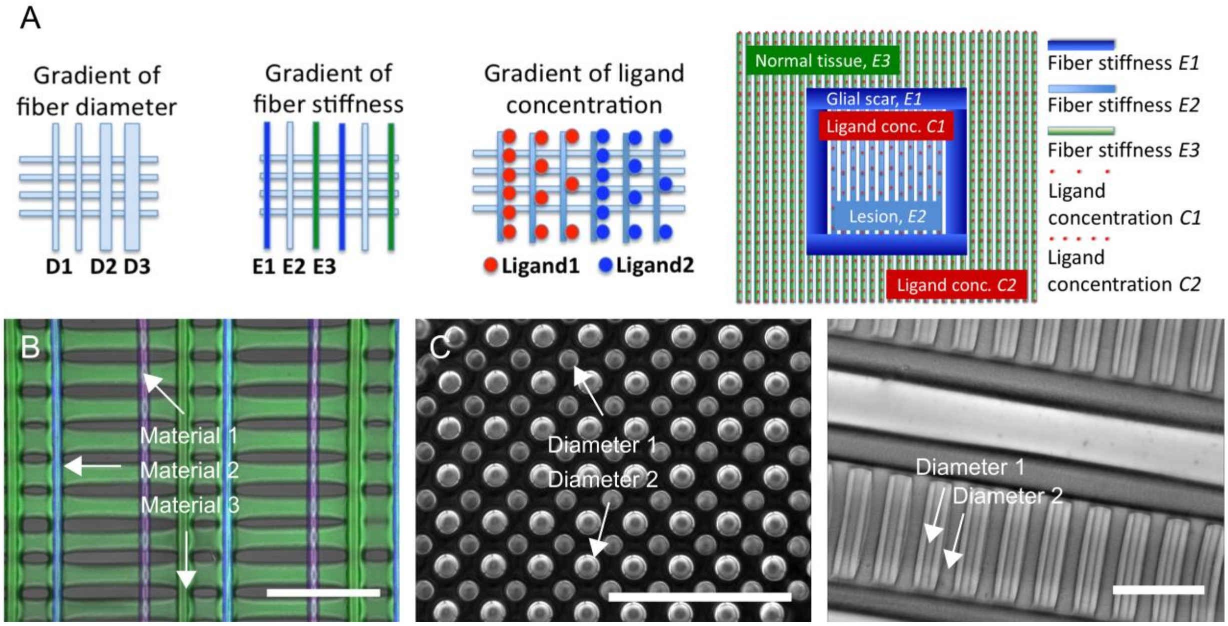

Figure S4. (A) Example schematics of different gradients mimicking *in vivo* environment (axon diameter, axon stiffness, ligand concentration) and hypothetical model of demyelinating lesion (right), incorporating different material stiffness and ligand concentrations, that can be fabricated using our materials and fabrication methods. (B) Poly-HEMA fiber bundle consisting of three distinct fiber inks corresponding to different stiffness (green = Ink 0; blue = Ink 1; purple = Ink 2; see Supplementary Fig. S6 for recipes). (C) Poly(HDDA-*co*-starPEG) pillar (left) and fiber (right) arrangements consisting of two diameters. Spatial heterogeneity can be achieved with PµSL via digital mask modification; multimaterial fabrication capability may be incorporated by flooding the resin bath with different photopolymer resins. Scale bars are 100 µm.

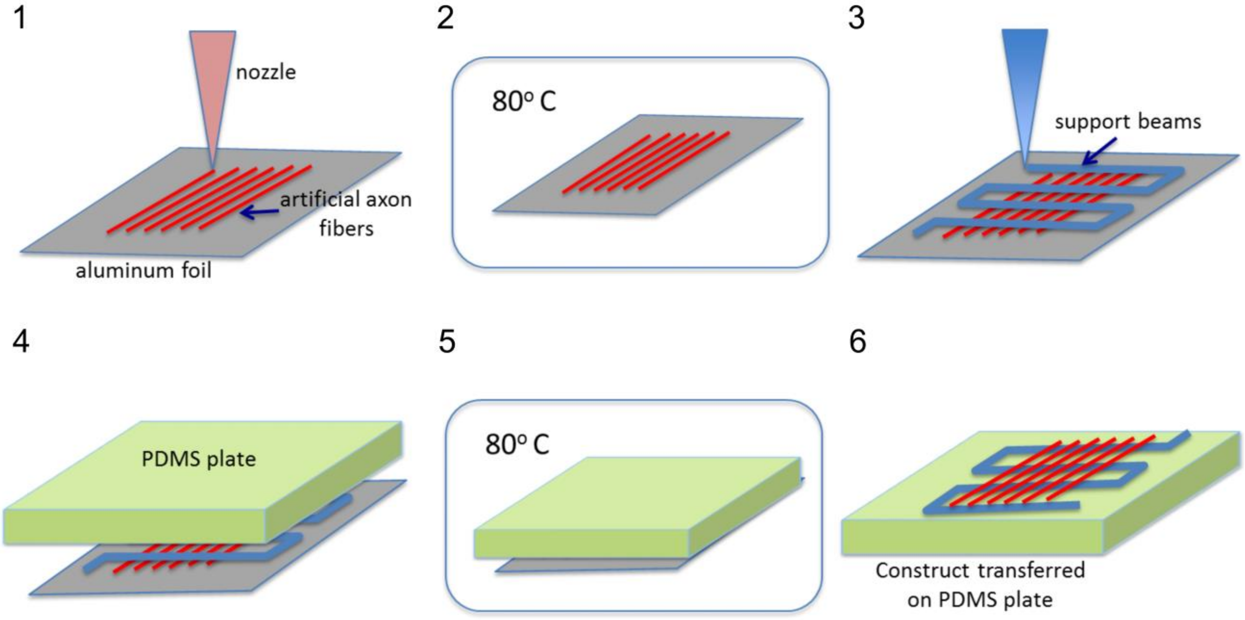

Figure S5. PDMS fiber printing steps. (1) Fibers with 10 µm diameter are printed first on the flat layer of aluminum foil. (2) Printed fibers are procured at 80° C to stabilize the shape. (3) Layer of supporting beams with 200 µm diameters is printed on top of the procured fibers. (4) The PDMS (or glass) plate is sandwiched on top of the supporting beams. (5) Construct is cured at 80° C. (6) Cured construct is removed from the oven and aluminum foil is removed from the fiber layer. The overhanging fibers on support beams are transferred onto PDMS (or glass) support.

A

Component	Ink 0 (wt%)	Ink 1 (wt%)	Ink 2 (wt%)
pHEMA (1 MDa)	10	10	10
pHEMA (300 kDa)	25	25	25
HEMA monomer	40	5	10
Water	23.7	33.7	33.7
Ethanol	0	25	20
EGDMA (comonomer)	1	1	1
Irgacure (initiator)	0.3	0.3	0.3

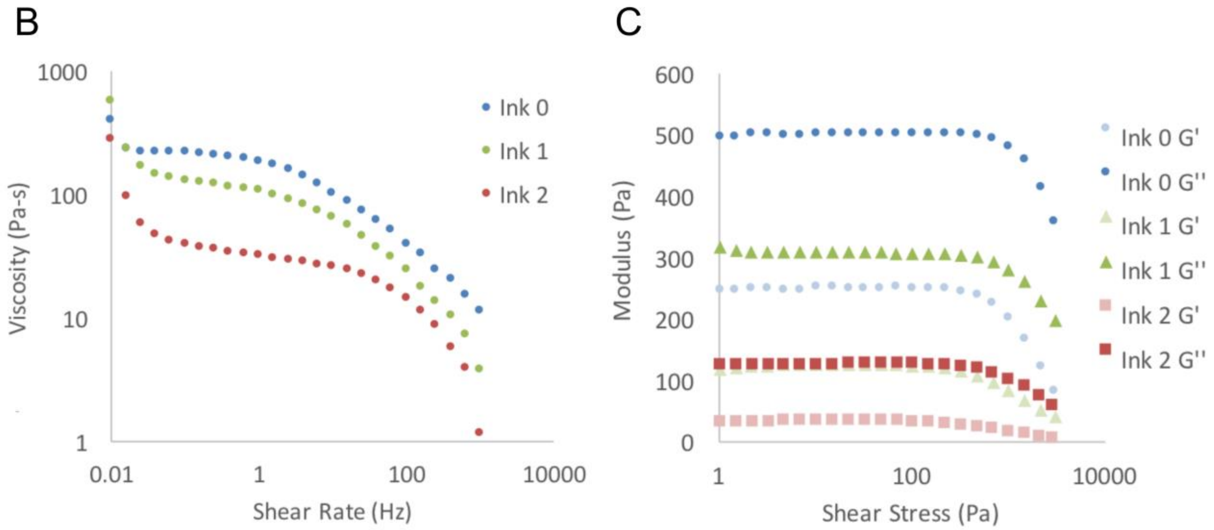

Figure S6. Viscoelastic properties of pHEMA inks and ink recipes. (A) Composition of pHEMA Inks printed in Supplementary Fig. S4. (B) pHEMA inks exhibit viscosity drop with increasing shear rate, (C) and shear thinning behavior at high shear stress.

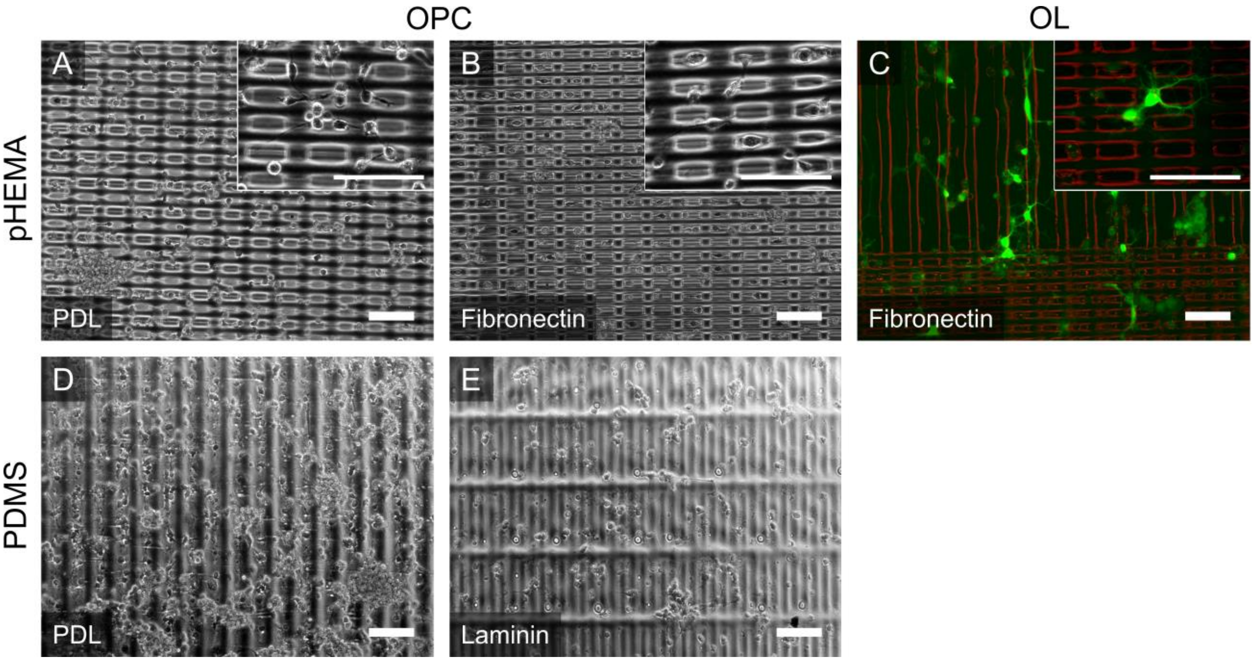

Figure S7. Additional demonstrations of oligodendrocyte (OL) and oligodendrocyte progenitor cell (OPC) compatibility with PDMS and pHEMA artificial axons coated with PDL, laminin and fibronectin. OPCs display simple bipolar morphology on pHEMA-based artificial axons coated with (A) PDL and (B) fibronectin, and PDMS-based artificial axons coated with (D) PDL and (E) laminin. (C) OPCs also differentiated into MBP-GFP expressing oligodendrocytes (green) that interacted with pHEMA-based artificial axons coated with fibronectin. Scale bars = 100 µm.

Movie S1. Migration of oligodendrocyte precursors (OPCs) on pHEMA artificial axons ("pHEMA-high-E" ink, stiffness ~333 kPa, 10 µm fiber diameter, day 1 in culture, poly-D-lysine coating). Time lapse: 1 h with 3 min interval; 37° C, 5% CO_2; phase contrast. Magnification 40x, acceleration in the movie 1200x.

Movies S2 and S3. 3D animation of confocal z-stacks of oligodendrocytes fully wrapping artificial axons ("HDDA-starPEG-high-E", stiffness ~140 kPa, 10 µm fiber diameter, day 20 in culture, poly-D-lysine coating). Fluorescence: red – artificial axon fiber, blue – Hoechst stained nuclei, green – MBP. Movies were generated using Imaris software (Bitplane).

Movies S4 and S5. Differentiation and wrapping of MBP-positive membrane by oligodendrocytes on pHEMA artificial axons ("pHEMA-high-E" ink, stiffness ~333 kPa, 10 µm fiber diameter, day 3 in culture, poly-D-lysine coating). Time lapse: 3 h with 3 min interval; 37° C, 5% CO_2. Magnification 40x, acceleration in the movie is 1200x. Red – artificial axon fibers (pseudo-color in phase contrast channel), green – fluorescence of MBP-GFP expressing oligodendrocytes.

Brief discussion of prior results for flat polymers and CNS microenvironment stiffness

Figure 5G in this manuscript demonstrates threefold increase in the percentage of wrapped artificial axons for printed material stiffness of E = 140 kPa as compared to E = 0.4 kPa. This should not be interpreted as "oligodendrocytes wrap stiffer materials preferentially," but rather considered in the context of this specific *magnitude* of elastic moduli and for the particular fiber diameters (15-20 μm) and surface coatings (laminin). Each of these variables can play a role in cell response, and this study was not designed or intended to compare the relative contributions of such physical properties in oligodendrocyte responses such as extent of wrapping.

The artificial axons herein are representing the geometry, stiffness and some surface ligands (laminin) or biological axons and are not intended to represent the structure and stiffness of an extracellular matrix scaffold. However, other configurations of 3D-printed, compliant polymers could be used to approximate the CNS tissue environment in this way. For such uses of a 3D-printed extracellular environment, it is useful to note that the above results are also in agreement with our previous findings on flat polyacrylamide-based hydrogel substrates, for which oligodendrocyte differentiation as quantified by MBP expression increased with increasing substratum stiffness over the range E = 0.1 to 70 kPa[23]. The stiffness of CNS tissue is approximated to be in the range E = 0.1 – 1 kPa, when measured on murine brain tissue slices via atomic force microscope-enabled indentation[15]. That finding was used to suggest that changes in mechanical stiffness of the local CNS environment of the cells may affect oligodendrocyte differentiation and myelination. Such mechanical changes may be related to either normal stiffness variation or pathological stiffness changes such as those observed in demyelinating or post-injury CNS lesions (reduced stiffness)[45,59] or brain tumors (increased stiffness)[60].

We are not aware of data demonstrating conclusively that the stiffness of axons themselves vary with disease state. Thus, in the context of this manuscript, our motivation was to better approximate the stiffness of a biological axon, which is much less stiff than most printed or electrospun fibers and materials currently used to assay myelination.

References 15, 23, 42, 45, and 59 in the manuscript are also cited in this Supplementary Information.
